# Robotic versus laparoscopic gastrectomy with lymph node dissection for gastric cancer: study protocol for a randomized controlled trial

**DOI:** 10.1186/s13063-018-2810-5

**Published:** 2018-07-31

**Authors:** Toshiyasu Ojima, Masaki Nakamura, Mikihito Nakamori, Keiji Hayata, Masahiro Katsuda, Junya Kitadani, Shimpei Maruoka, Toshio Shimokawa, Hiroki Yamaue

**Affiliations:** 1 Second Department of Surgery, School of Medicine, Wakayama Medical University, 811-1, Kimiidera, Wakayama, 641-8510 Japan; 2 Clinical Study Center, School of Medicine, Wakayama Medical University, Wakayama, Japan

**Keywords:** Gastric cancer, Laparoscopic gastrectomy, Robotic gastrectomy, Minimally invasive surgery, Phase III clinical trial

## Abstract

**Background:**

Laparoscopic gastrectomy (LG) has several benefits as a treatment of gastric cancer (GC), including reduced pain, early recovery of intestinal function, and shorter hospital stay. LG still has several drawbacks, however, including limited range of movement, amplification of hand tremors, and inconvenient surgical positioning. Around the peripancreatic area, laparoscopic lymph node dissection, therefore, remains challenging; postoperative pancreatic fistula occurs in around 4–7% of patients undergoing LG. Robotic surgery, on the other hand, plays a role in ergonomics and offers several advantages, including 7° of wrist-like motion, less fatigue, tremor filtering, motion scaling, and three-dimensional vision. In our previous retrospective study, we compared the safety and feasibility of surgical outcomes of LG and robotic gastrectomy (RG) for patients with GC. In our previous results, in the LG group, intra-abdominal infectious complications were found in 11%. In the RG group, however, none were found. Our RG procedure may be associated with decreased incidence of intra-abdominal infectious complications. Prospective randomized controlled trials (RCTs) comparing LG and RG are required, however. We begin an RCT to compare short-term surgical and long-term oncological outcomes of LG and RG for GC patients.

**Methods:**

This is a randomized, single-center clinical trial. All included patients are adults with primary carcinoma of the stomach, in whom the tumor is considered surgically resectable (stages I–III). Included in this trial are 240 patients with GC. The primary endpoint is to assess the incidence of postoperative intra-abdominal infectious complications including pancreatic fistula, intra-abdominal abscess, and anastomotic leakage. Secondary endpoints include the incidence of any complications (both related and unrelated to surgery), surgical results, postoperative course, and oncological outcomes.

**Discussion:**

Although its short-term outcomes have been proven comparable to LG in comparative studies, use of RG remains restricted, partly due to the lack of informative RCTs pertaining to it. To evaluate the surgical and oncological outcomes of RG, we therefore undertake a prospective RCT. The obtained results will be useful for reducing the restrictions and for adaptive expansion of RG for patients with GC.

**Trial registration:**

University Hospital Medical Information Network Clinical Trials Registry, ID: UMIN000031536. Registered on 1 March 2017.

**Electronic supplementary material:**

The online version of this article (10.1186/s13063-018-2810-5) contains supplementary material, which is available to authorized users.

## Background

Since laparoscopic gastrectomy (LG) with radical lymphadenectomy for gastric cancer (GC) was developed in 1991, it has been widely accepted as a less invasive procedure than open gastrectomy [1, 2]. LG has several benefits for GC patients, such as reduced pain, early recovery of intestinal function, and shorter hospital stay [2–4]. LG still has several drawbacks, however, including the limited range of movement, amplification of hand tremors, and inconvenient surgical positioning. Laparoscopic lymph node dissection around the peripancreatic area, such as the suprapancreatic or infrapyloric lymph nodes, remains challenging. Indeed, postoperative pancreatic fistula occurs in around 4–7% of patients undergoing LG [5, 6].

Robotic gastrectomy (RG) was developed in 2000 as an alternative, minimally invasive approach that may overcome the drawbacks of LG. It plays an essential role in ergonomics and offers advantages, such as 7° of wrist-like motion, less fatigue, tremor filtering, motion scaling, and three-dimensional vision [7, 8]. We propose that this innovative technology can overcome some limitations of LG, and, as a result, the incidence of morbidity can be reduced by RG.

In our previous retrospective study, we compared the safety and feasibility of surgical outcomes of LG and RG for patients with GC (manuscript under consideration). Since RG has fewer samples than LG, overall postoperative complication rates were comparable between the groups (Table 1).

**Table 1:** Preliminary data (January 2011 to December 2017)

	Laparoscopic group	Robotic group
Number of patients	879	20
Age, years, median (range)	70 (26–96)	71 (47–85)
Gender: male / female	607 / 272	13 / 7
TNM, 8th ed, stages I / II / III / IV	640 / 72 / 125 / 42	18 / 1 / 1 / 0

Intra-abdominal infectious complications, were found in 11% of the LG group, including pancreatic fistula, abscess, and anastomotic leakage higher than Clavien-Dindo grade II [9]. In the RG group, however, they were not found. Our RG procedure may, therefore, be associated with decreased incidence of intra-abdominal infectious complications.

RG also results in decreased morbidity compared with LG, according to other retrospective studies [10–12]. Prospective randomized controlled trials (RCTs) comparing LG and RG are required, however. This RCT compares short-term surgical and long-term oncological outcomes of LG and RG for GC.

## Methods

### Study objectives

This prospective, single-center, phase III trial aims to demonstrate the benefits of RG over LG for resectable gastric cancer regarding the reduction of complications.

### Study setting

A single-institution, randomized, phase III study.

### Endpoints

The incidence of postoperative intra-abdominal infectious complications is analyzed and assessed, including pancreatic fistula, intra-abdominal abscess, and anastomotic leakage, according to the Clavien-Dindo classification. Complications higher than grade II (requiring pharmacological treatment with drugs) are regarded as clinically significant [9]. Secondary endpoints are: (1) incidence of any complications (both related and unrelated to surgery) higher than Clavien-Dindo grade II; (2) surgical results, such as operation time, blood loss, transition rate to open or laparoscopic surgery, and the number of harvested lymph nodes; (3) postoperative course; such as time to start of drinking, time to start of eating, postoperative hospital stay, and weight loss rate; and (4) oncological outcomes; such as overall survival (OS) and relapse-free survival (RFS). OS duration is defined as days from operation. RFS duration is defined as days from operation to relapse. Patients are followed-up for 5 years after surgery.

### Sample size

Determination of the rate of postoperative intra-abdominal infectious complications is the primary endpoint of this trial. The sample size to predict the number of patients necessary for statistical validity (two-sided test) is based on our retrospective data from between January 2011 and December 2017 (*n* = 899) (Table 1). According to this data, the incidence rate of intra-abdominal infectious complications after LG was 11%. Therefore, the incidence rate of 11% in the LG group was estimated. As this incidence rate in the RG group was 0% in the retrospective study, 2% was expected to be the incidence rate of intra-abdominal infectious complications in the RG group (odds ratio = 0.165). We calculated that 117 patients are required in each arm of this study with a significance *α* = 0.05 and a power of (1 − *β*) = 0.8. Anticipating follow-up loss, we calculated that 120 patients are required in each arm of this study, a total study population of 240 patients.

### Eligibility criteria

Before participation in this study, patients undergo esophagogastroscopy and enhanced computed tomography (CT) scans of the chest and abdomen to evaluate the pretreatment tumor stage.

#### Inclusion criteria

(i) Histologically proven gastric carcinoma
(ii) Resectable gastric cancer according to the TNM classification (clinical stages I–III) [13]
(iii) Not applicable for endoscopic submucosal dissection according to the Japanese classification [14]
(iv) Aged between 20 and 90 years
(v) Performance status (ECOG) 0 or 1
(vi) Body Mass Index of < 35
(vii) No history of gastrointestinal surgery
(viii) No history of chemotherapy or radiotherapy
(ix) Normal function of the major organs; indicated by a leukocyte count of over 3000 mm^3^, a platelet count of over 100,000 mm^3^, aspartate aminotransferase and alanine aminotransferase levels less than 200 IU/L and creatinine levels less than 2 mg/dL
(x) Proven written informed consent

#### Exclusion criteria

(i) Synchronous or metachronous malignancies other than carcinoma in situ
(ii) Pregnant or breast-feeding
(iii) Severe mental illness
(iv) Continuous systemic steroid therapy
(v) History of myocardial infarction or unstable angina pectoris within 6 months
(vi) Uncontrollable hypertension
(vii) Uncontrollable diabetes mellitus or administration of insulin
(viii) Respiratory disease requiring continuous oxygen therapy
(ix) History of deep vein thrombosis

### Participating surgeons

The complication rate can be a result of the experience of the operating surgeon, which might bias results. To prevent surgeon bias, participating surgeons satisfy the following criteria: (1) having experience of more than 40 LG, (2) having experience of more than 20 RG, (3) being a qualified surgeon according to the endoscopic surgical skill qualification system, Japan Society for Endoscopic Surgery, and (4) being a board-certified Fellow of the Japanese Society of Gastroenterological Surgery.

LG is performed by three surgeons, (TO, MiN, and MaN) and RG is performed by one surgeon (TO). These clinicians each fulfill requirements for participation, and have sufficient training in dry and wet laboratories.

### Randomization

**Fig. 1:**
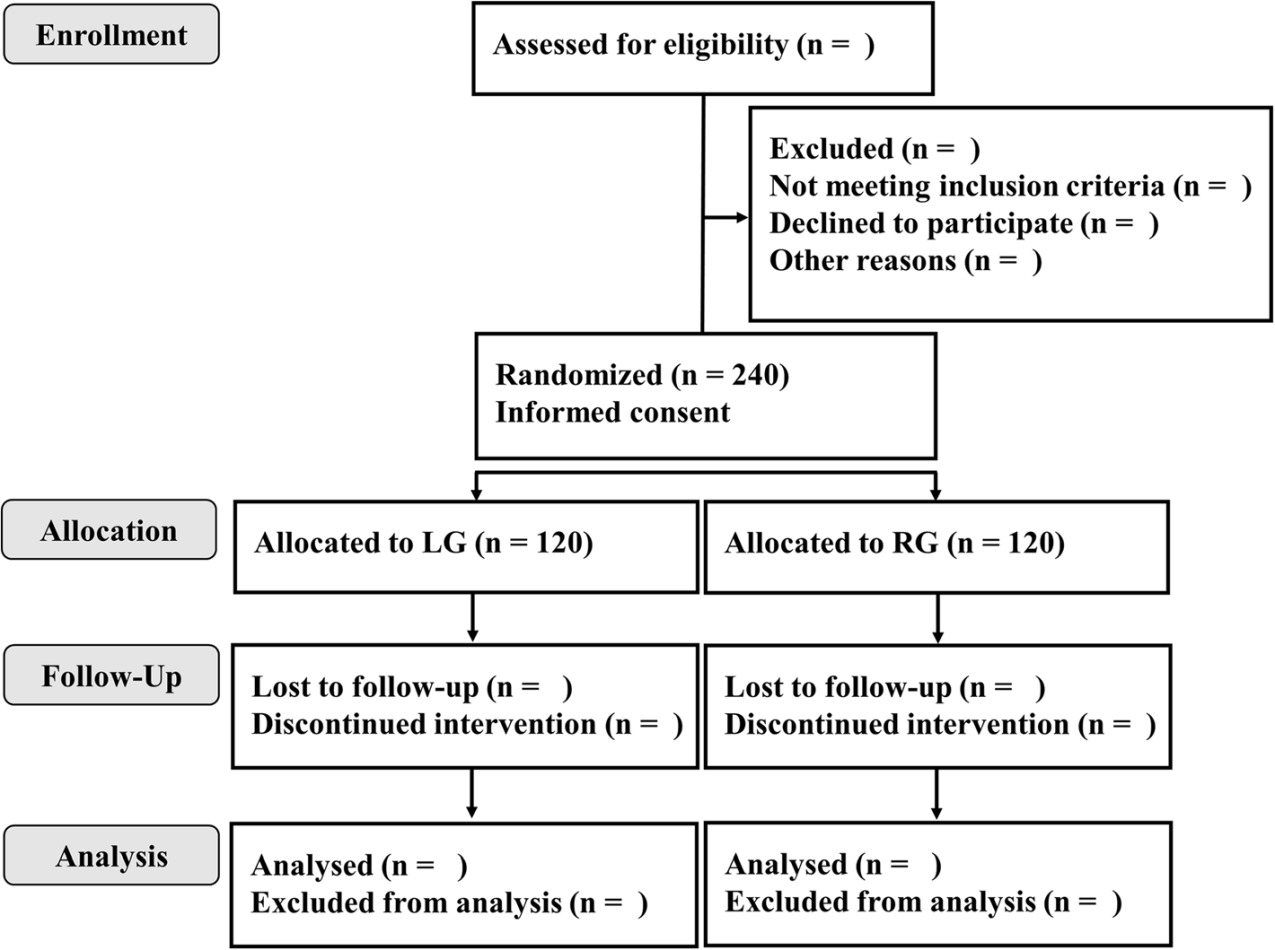
Consolidated Standards of Reporting Trials (CONSORT) flow diagram

After confirmation of the eligibility criteria, registration is made by telephone to the central registry in Wakayama Medical University Hospital (WMUH). Study in each group is carried out using a series of consecutive numbers assigned by the WMUH central registry. Patients are randomized to either the LG arm or RG arm by a minimization method balancing the arms by gastrectomy type (distal gastrectomy / total or proximal gastrectomy) (Fig. 1).

### Data collection and statistics

**Fig. 2:**
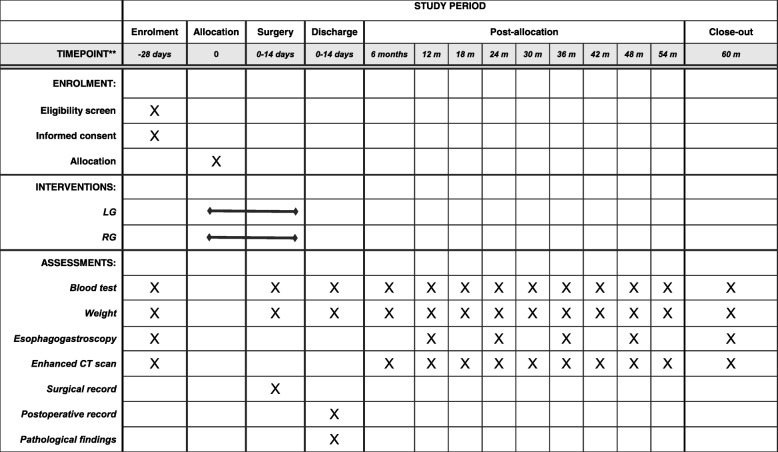
Standard Protocol Items: Recommendations for Interventional Trials (SPIRIT) Figure

Data is collected prospectively for all patients including history, physical examination, laboratory data, pathologic examination, perioperative clinical information and complications (Fig. 2). Data is collected via datasheets on paper and kept securely. All handling cases are managed by subject identification code or anonymized registration number. The correspondence table of the anonymizing code and names and the consent form containing the names are kept strictly in the separate lockable document storage at WMUH. All required parameters are collected in an SPSS data file (SPSS version 25, IBM statistics, Chicago, IL, USA).

### Interim analysis and monitoring

To ensure the safety and feasibility of this study, it consists of two stages (the first half and the latter half, each with 120 patients). If safety and feasibility are confirmed in the first half from the perspective of an independent outsider (Data Monitoring Committee), the 121st patient and beyond are enrolled in the latter half. In-house monitoring is performed every year by a third party to evaluate and improve the progress and quality of the study. Stoppage of the trial may also be recommended in case of insufficient enrollment or overwhelming/inferior efficacy.

### Surgical technique

#### Laparoscopic gastrectomy with lymph node dissection

Details of the LG procedures performed at WMUH have been previously described [4, 15]. The basic extent of lymph node dissection in the present series is D1+ or D2 [14]. The greater omentum is resected up to the inferior portion of the spleen using a laparoscopic, ultrasonically activated device (USAD) and the harmonic scalpel™ (EthiconEndo-Surgery, Cincinnati, OH, USA). The left gastroepiploic vessels are dissected at the point before the first branch (nos. 4d, 4sb). After completion of omentectomy, using the USAD, the root of the right gastroepiploic vein and artery are isolated and transected (no. 6). The root of the right gastric artery is isolated in the hepatoduodenal ligament and transected (no. 5). The lesser omentum along the liver edge to the esophagogastric junction is resected. The perigastric lymph nodes are dissected along the upper lesser curvature up to the esophagogastric junction (nos. 1 and 3). For laparoscopic D1+ lymphadenectomy, the lymph nodes around the celiac trunk (no. 9) are dissected, and the root of the left gastric vein and artery are isolated and transected using USAD (no. 7), and successively, the lymph nodes along the common hepatic artery are dissected (no. 8a). For laparoscopic D2 lymph node dissection, the lymph nodes along the proper hepatic artery (no. 12a) and along the splenic artery (no. 11) are also dissected. Lymph node dissection is completed intra-corporeally.

#### Robotic gastrectomy with lymph node dissection

All RG procedures are performed using the da Vinci™ Si or Xi Surgical System (Intuitive, Sunnyvale, CA, USA) with four articulating robotic arms; a central arm for a 30° rigid endoscope, a first arm for monopolar scissors, and a second arm for fenestrated bipolar forceps, and a third arm for Cadiere forceps, respectively [10–12, 16]. One additional port for assisting forceps is placed at the right umbilical level. The RG procedure is not different from the LG procedure with D1+ or D2 lymph node dissection as described above.

#### Reconstruction

In both the LG and RG groups, intra-corporeal anastomosis using linear staplers, such as gastroduodenostomy, gastrojejunostomy, or esophagojejunostomy is performed. A single abdominal drain is inserted into the left subphrenic cavity after reconstruction in both groups.

#### Postoperative management

Patients are given antibiotics twice only during surgery. The nasogastric tube is removed when the patient is awoken from anesthesia. Postoperative pain control consists of patient-controlled analgesia. Patients are encouraged to be out of bed and walking around the ward under the guidance of a physiotherapist or nurse the day after surgery. Patients will be discharged when they can pass stools, are able to drink, can walk, and are comfortable with orally administered analgesia.

#### Follow-up

Adjuvant chemotherapy with S-1, an oral anticancer drug, for 1 year is included in protocol treatment for patients with pathological stages II–III gastric cancers. Different regimens are not used. All patients are followed up for 5 years or until death. Enhanced CT scans of the chest and abdomen are evaluated every 6 months; esophagogastroscopy is evaluated every year during follow-up (Fig. 2). The study protocol adheres to the SPIRIT statement (Additional file 1).

## Discussion

Our retrospective study, and others comparing LG and RG, show that RG is a safe and feasible alternative to LG regarding short-term surgical outcomes (10–12). Nonetheless, RG is still presently restricted, is in part to due to the lack of relevant RCTs pertaining to it. Therefore, to evaluate the surgical and oncological outcomes of RG, we proceed with a prospective RCT. The results obtained should be useful for improvement of the status and adaptive expansion of RG for GC patients.

In the sample size calculation, we assume a difference in intra-abdominal infectious complications of 11% in LG versus 2% in the RG group. These estimates were calculated according to our preliminary data of 899 patients (Table 1). In previous non-randomized study with 526 patients, the rates of infectious complications were significantly greater in the LG than in the RG group (11.4% versus 2.3%) (11). These values are equal to our predicted values. Therefore, we believe that our predicted values can be realized.

This study has several limitations. It is conducted at a single institution. Due to the small sample size, findings from this trial do not allow established clinical application, but rather serve to inform the need for larger multicenter, phase III, RCTs on RG for GC.

### Trial status

WMUH Institutional Review Board approved the final version of the protocol prior to the start of the study (approval number: 2283). It was registered on the University Hospital Medical Information Network Clinical Trials Registry (UMIN000031536). The trial is open for recruitment from April 2018.

## Additional file

Additional file 1: Standard Protocol Items: Recommendations for Interventional Trials (SPIRIT) 2013 Checklist: recommended items to address in a clinical trial protocol and related documents*. (DOC 121 kb)

## Abbreviations

CT: Computed tomography
GC: Gastric cancer
LG: Laparoscopic gastrectomy
OS: Overall survival
RCT: Randomized controlled trial
RFS: Relapse-free survival
RG: Robotic gastrectomy
USAD: Ultrasonically activated device
WMUH: Wakayama Medical University Hospital
